# Biomimetic platelet-camouflaged drug-loaded polypyrrole for the precise targeted antithrombotic therapy

**DOI:** 10.1186/s12951-023-02197-3

**Published:** 2023-11-22

**Authors:** Zhining Zhao, Xiaodan Li, Yan Wang, Cheng Liu, Guixia Ling, Peng Zhang

**Affiliations:** Shenyang Pharmaceutical University, 103 Wenhua Road, Shenyang, 110016 China

**Keywords:** Platelet membrane, Anticoagulation, Photothermal thrombolysis, LEDVT

## Abstract

Lower extremity deep venous thrombosis (LEDVT) affects patient’s quality of life for a long time, and even causes pulmonary embolism, which threatens human health. Current anticoagulant drugs in clinical treatment are hampered by the risk of bleeding due to poor targeting and low drug penetration. Here, we used platelet (PLT)-like biological targeting to enhance the delivery and accumulation of nanomedicines in thrombus and reduce the risk of bleeding. Meanwhile, the parallel strategy of “thrombus thermal ablation and anticoagulation” was applied to increase the permeability of drugs in thrombus and achieve the optimal antithrombotic effect. Polypyrrole (PPy) and rivaroxban (Riv, an anticoagulant drug) were co-assembled into platelet membrane-coated nanoparticles (NPs), PLT-PPy/Riv NPs, which actively targeted the thrombotic lesion at multiple targets in the platelet membrane and were thermally and drug-specific thrombolysed by 808 nm laser irradiation. The combination therapy resulted in up to 90% thrombolysis in a femoral vein thrombosis model compared to single phototherapy or drug therapy. The results showed that the nanoformulation provided a new direction for remote precise and controlled sustained thrombolysis, which was in line with the trend of nanomedicine towards clinical translation.

## Introduction

Thrombosis-related diseases such as ischemic stroke, acute myocardial infarction and deep vein thrombosis remain the leading causes of mortality or morbidity worldwide, far exceeding the incidence of tumors [1]. Lower extremity deep venous thrombosis (LEDVT) is a disease of returning obstacles of the lower limb vein. If not treated in time, it will affect the life quality of patients for a long time, and may be complicated with pulmonary embolism, which eventually endanger life. Currently, surgical intervention and drug therapy are the main clinical treatment methods. Due to the high cost and invasiveness of surgery, drug thrombolytic therapy remains the predominant choice for clinical thrombosis treatment. Drug therapy includes thrombolytic therapy, anticoagulant therapy and antiplatelet therapy, among which anticoagulant strategies remain the mainstay of LEDVT treatment and prevention [2–4]. However, the therapeutic drugs are easily cleared by the liver, leading to shortened circulation time. Meanwhile, off-target effect of drugs is accompanied by serious bleeding risk and other problems [5]. Therefore, how to improve the targeting and permeability of drugs at the thrombosis site, reduce the immunogenicity of the organism and reduce the hemorrhagic risk of thrombolytic drugs have become the primary problem to be solved in drug therapy.

It has been reported that photothermal action can improve the permeability of drugs [6–9]. The principle is that the non-covalent interaction in the thrombus is disrupted by photothermal heating, leading to loosening and shrinking of the thrombus. The shear force of the arterial or venous fluid will disperse the loose clot, resulting in low concentration of local polymeric fibrin, which alleviates the obstruction and promotes the penetration of drugs in the thrombus. Targeted photothermal therapy (PTT) is highly selective and can minimize the damage to the targeted region, providing a new therapeutic strategy for thrombolytic therapy.

Polypyrrole (PPy) is an excellent photothermal material with good biocompatibility and non-toxicity, which has been used in the targeted therapy of thrombolytic [10, 11]. Currently, it has been reported that PPy-based thrombus-targeting agents utilize electrostatic action to use heparin as a chemically targeted ligand to achieve active targeting of thrombus [12]. However, these nanoparticles (NPs) are exogenous and susceptible to recognition by the body’s immune system, leading to the problem of premature clearance of antibodies against these NPs, while single-target modifications face the risk of being easily off-target. Currently, platelet membranes have been proved to be a potential application in antithrombotic therapy as a nano-carrier due to its unique targeting and precise drug delivery ability [13]. At present, it is reported in the available literature that platelet membranes were successfully coated on the surface of NPs by gradient centrifugation and ultrasonic co-extrusion. The successful coating of platelet membranes and the presence of related biomarkers were observed, with most of the platelet membrane proteins remaining on the surface of the NPs [14–16]. However, the removal of the internal proteins during the platelet extraction stage might result in the failure to activate downstream signal pathways in platelets [17, 18]. Thus, the presence of these membrane proteins enabled NPs to recognize activated platelets and bind thrombi but not induce platelet activation and aggregation. It is found that platelet membranes can target activated platelets but have no ability to activate platelets. At the same time, they interact with activated platelets in multi-targeted way, mainly by combination with P-selectin and GP IIb/IIIa on platelet membranes [17, 19]. Therefore, platelet membranes become the excellent nanocarriers for antithrombotic therapy. Next researchers untilized bionic platelet membranes as thrombotic nano-targeting carriers with both encapsulated PPy NPs and free heparin to achieve precise targeting of thrombosis [20]. Although this nano-preparation avoided the immunogenicity of the organism and improved the targeting, it faced a problem of easy leakage of the drug. Based on the above ideas, core PPy NPs with both photothermal and drug loading properties were designed to provide a new idea for bionic drug delivery.

**Fig. 1:**
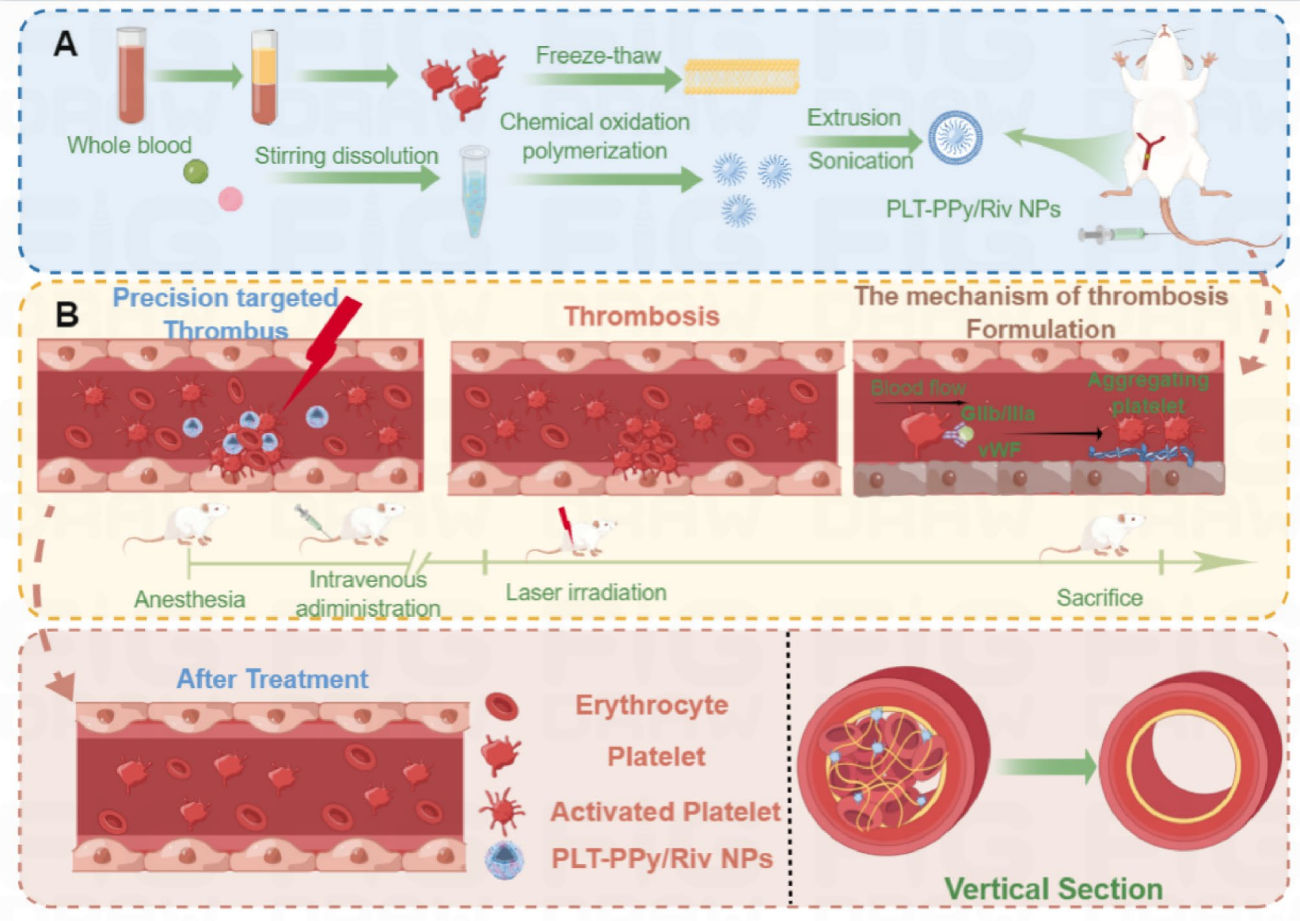
Schematic diagram of the preparation of PLT-PPy/Riv NPs and precise targeting of LEDVT. **a** Synthesis process of PLT-PPy/Riv NPs. **b** PLT-PPy/Riv NPs achieved Riv enrichment in FeCl_3_-induced LEDVT and showed good antithrombotic properties with the help of platelet targeting characteristics

Platelet membrane as a “bullet shell” was used for the first time and a polymer with high photothermal conversion efficiency (PCE) was loaded as the “ammunition” to actively target the thrombus site, becoming a novel antithrombotic treatment strategy. The “ammunition” assembled into core NPs by the intermolecular interaction between pyrrole and the anticoagulant rivaroxban (Riv) retained the original PCE of PPy (Fig. 1). The 808 nm laser irradiation after 75 min intravenous injection not only directly ablated the thrombus, but also promoted the release of Riv in PLT-PPy/Riv NPs for therapeutic purposes. The targeted antithrombotic effect of different nano-formulations was evaluated in vitro and vivo. The results showed that the designed PLT-PPy/Riv NPs had the optimal thrombolytic effect among all groups. The photothermal effect accelerated the lysis of the clot and improved the thrombolytic effect while reducing side effects such as bleeding, overcoming the shortcomings of existing therapy. All the results proved the great potential of PLT-PPy/Riv NPs in the treatment of thrombosis.

## Materials and motheds

### Materials

Riv was purchased from Shanghai Yuanye Biotechnology Co., Ltd. Pyrrole, thrombin, prostaglandin 1 (PGE1) and protease inhibitor (PMSF) were purchased from Shanghai Maclean Biochemical Technology Co., Ltd. Ferric chloride hexahydrate, ammonium persulfate, sodium dodecyl sulfate (SDS), calcium chloride were purchased from Tianjin Hengxing Chemical Reagent Co., Ltd. 1,1′-dioctadecyl-3,3,3′,3′-tetramethylindodicarbocyanine, 4-chlorobenzenesulfonate salt dye (DiD dye), Calcein-AM dye, phosphate buffered saline (PBS) and fetal bovine serum (FBS) were purchased from Meron Biotechnology Co.,Ltd. The used water was deionized water and all experimental chemicals were of reagent grade. All animal experiments were approved by the Animal Center of Shenyang Pharmaceutical University. Sprague-Dawley rats (SD rats) (180–220 g, males) and Kunming mice (KM mice) (18–22 g, males) were from ChangSheng Biotechnology Company Limited (Liaoning, China), which were raised in SPF environment.

### Preparation of NPs

According to a previously reported method for rat platelet purification, the orbited whole blood of SD rats was collected into the collection vessels containing anticoagulants by capillary method. Platelet-rich plasma (PRP) was obtained by centrifugation at 200 g for 10 min at room temperature. An equal volume of ACD solution (acid-citrate-dextrose) was added and centrifuged at 100 g for 10 min to remove the remaining erythrocytes [15]. After centrifugation at 800 g for 10 min, the supernatant was discarded and the platelets were resuspended in PBS containing 2 μM PGE1 and PMSF. These were repeated freeze-thaw cycles for three times, centrifuged at 4000 rpm for 4 min, and washed three times with PBS containing PMSF. Finally, the platelet membranes were resuspended in 0.2 mM EDTA aqueous solution and stored at -80℃ for further use.

0.3 g of SDS was weighed and dissolved in 5 mL of deionized water, and 75 μL of pyrrole monomer and about 4 mg of Riv were added. The solution was stirred to dissolve completely, and then 0.0492 g of ammonium persulfate was added to oxidize and polymerize for 2 h to form SDS-PPy/Riv NPs. Subsequently, the obtained platelet membranes were co-sonicated with NPs in an ice bath for 20 min and then the extruder of 200 nm polycarbonate membrane was used to extruder 10 times to remove the excess platelet membrane to obtain PLT-PPy/Riv NPs with uniform particle size. The PLT-PPy NPs were prepared as above without the addition of Riv.

### Characterization of nano-formulations

The size, polydispersity index, and zeta potential of SDS-PPy/Riv NPs and PLT-PPy/Riv NPs were measured by Zetasizer. The morphological changes of nanoformulations (SDS-PPy/Riv NPs and PLT-PPy/Riv NPs) were characterized by transmission electron microscopy (TEM) after negative staining with sodium phosphotungstic acid solution. We used an ultrafiltration method to investigate the leakage of Riv from nano-formulations in a physiological environment to evaluate the stability of the NPs in vitro. According to a previous report, the composition of FBS was very similar to that of body fluids [21], and PBS (pH 7.4) containing 10% FBS was often used in stability experiments to simulate the medium of the physiological environment [22]. Briefly, we incubated PLT-PPy/Riv NPs in PBS (pH 7.4) containing 10% FBS for 12 h at 37 ℃. The samples were placed in ultrafiltration tube with a cut-off molecular weight of 30 kDa, and centrifuged (4000 rpm, 10 min), and the concentration of Riv in the filtrate was determined by HPLC at pre-set time intervals. Finally, in order to explore the action mechanism of the core polymer, we used Chemdraw and Materials studio to conduct molecular docking and screen the degree of pyrrole polymerization at the optimal binding energy of PPy and Riv.

### In vitro light-triggered Riv release

The release behavior of Riv in PLT-PPy/Riv NPs was determined by dialysis method. PBS (pH = 7.4) containing 0.2% SDS was used as the release medium. The prepared NPs were placed in the dialysis bag with a molecular weight of 3500 Da and immersed in 100 mL of release medium. The laser irradiation group was irradiated with 808 nm (2.22 W/cm^2^) laser for 10 min before the release, and subsequently 2 mL sample was taken at predetermined time to determine the concentration of Riv by HPLC while supplementing an equal volume of the release medium solution. And then the cumulative release amount of Riv was calculated.

### In vitro photothermal performance of PLT-PPy/Riv NPs

#### PCE

To investigate the photothermal performance of the prepared formulations, PBS, SDS-PPy/Riv NPs, PLT-PPy NPs, and PLT-PPy/Riv NPs were irradiated with 808 nm laser respectively. The temperature changes and thermal imaging maps of different samples were recorded by infrared thermal imager during laser illumination. When the samples were found to warm up to a stable level, the irradiation was stopped. Meanwhile, in order to calculate the PCE of PLT-PPy/Riv NPs, we recorded the cooling change and fitted the cooling curve. The volume of each experiment was 1 mL. The calculation formula of the PCE of PLT-PPy/Riv NPs is as follows [23]:

$$\eta = \frac{{hs\Delta Tmax - Qs}}{{I(1 - {{10}^{ - A}})}}$$

$$\theta =\frac{T-Tsur}{Tmax-Tsur}$$

$${\rm{t = - \tau In\theta }}$$

$$hs=\frac{mc}{\tau }$$

In the equation, s is the surface area. h is the heat transfer coefficient. Tmax is the maximum temperature at equilibrium. Tsur is the ambient temperature. Qs is the heat absorption rate of water. I is the laser power density. A is the absorbance of the NPs at 808 nm, and τ is the time constant.

#### Photothermal stability

To evaluate the photothermal stability of PLT-PPy/Riv NPs, 1 mL PBS solution, Riv Sol, SDS-PPy/Riv NPs, PLT-PPy NPs and PLT-PPy/Riv NPs were respectively irradiated under 808 nm (2.2 W ·cm-2) laser for 10 min. The laser was turned off and cooled naturally. The cooling changes of PLT-PPy/Riv NPs over time were recorded and repeated 3 times.

### Assessment of target ability in vitro

#### In vitro study of activated platelet targeting ability

We diluted the DiD stock solution into the DiD working solution and incubated it with the obtained platelet membranes at 37 ℃ for 20 min. After incubation, the solution was centrifuged at 800 g for 3 min and washed continuously for several times to remove the excess DiD. Finally, the DiD-labeled cell membranes were resuspended with PMSF-containing PBS solution for standby. The DiD-labeled NPs were obtained by sonicating the DiD-labeled cell membranes with SDS-PPy/Riv NPs for 20 min and washing them several times by centrifugation in PBS. The DiD-labeled NPs were incubated with the extracted inactivated platelets (dissolved in PBS solution containing 30 ng/mL (PGE1) and activated platelets (dissolved in PBS solution containing 8 × 10^− 9^ M CaCl_2_ and 2 U/mL thrombin) for 30 min [16]. Then the solution was washed continuously with PBS to remove the free NPs, and the DiD-labeled NPs were resuspended in PBS for analysis by flow cytometry.

To further verify the adhesion of PLT-PPy/Riv NPs to activated platelets, confocal microscopy was used for visual analysis. Platelets extracted from fresh blood of rats (activated and inactivated groups) were labeled with Calcein-AM, and then co-incubated with DiD-labeled NPs for 45 min. And after the end of co-incubated, the platelets were washed three times by centrifugation at 250 g. Finally, they were resuspended with PBS solution, filmed, and observed by confocal observation.

#### In vitro targeting ability of clots

In order to further evaluate the in vitro targeting ability of the prepared formulations, we explored the targeting ability of PLT-coated NPs to the prepared artificial blood clots. Fresh whole blood of rats was collected by capillary orbital-blood extraction in microcentrifuge tubes with a final concentration of 1 U·mL^− 1^ thrombin and 2.5 mM CaCl_2_ solution, which was incubated at 37℃ for 1 h to obtain the softer artificial blood clots [12]. PBS, Riv solution (Riv Sol), SDS-PPy/Riv NPs, PLT-PPy NPs, and PLT-PPy/Riv NPs were incubated with artificial blood clots for 30 min, respectively, and the excess NPs were removed by washing with PBS. Finally, the fluorescence intensity was detected with in vivo imaging system (IVIS).

### In vitro thrombolysis studies

Here, we used the Drabkin method to assess the in vitro thrombolytic effect of different preparations. Then the in vitro antithrombotic test was carried out, and the above-prepared clots were randomly divided into five groups. After laser treatment with PBS, Riv Sol, SDS-PPy/Riv NPs, PLT-PPy NPs and PLT-PPy/Riv NPs (all the administration groups received equal doses of 0.9 mg/kg Riv), respectively, the equal volume of Drabkin’s reagent was added and placed at room temperature for 20 min. After centrifugation at 10,000 g for 10 min, the supernatant was taken and the OD value of cyanogenic hemoglobin was determined at 540 nm by using a microplate reader.

### Assessment of thrombolysis in vivo

#### Mouse thrombosis model and antithrombic effect

SD rats were anesthetized by intraperitoneal injection of chloral hydrate (10%). After the rats were supine and secured with tape, the fur was removed by using depilatory cream and an incision was made in the inner thigh to expose the femoral vessels. Then the femoral vessels were covered with filter paper (0.5 cm × 0.5 cm) saturated with 10% ferric chloride solution for 5 min to form femoral thrombosis. Residual ferric chloride was then removed by rinsing with saline. We randomly divided the SD rats of the thrombosis model into five groups: PBS group (control group), Riv Sol group (drug-only group), SDS-PPy/Riv NPs + laser group (photothermal + drug treatment group), PLT-PPy NPs + laser group (photothermal group only), PLT-PPy/Riv NPs + laser group (photothermal + drug-targeted treatment group) (all the drug-treated rats received an equal dose of 0.9 mg/Kg Riv). In the laser group, 808 nm laser irradiation was performed for 10 min after 75 min of tail vein injection, followed by wound suturing. After 24 h, the thrombus-containing femoral vessels were removed and immersed in 4% paraformaldehyde, then were embedded, sectioned, stained with hematoxylin and eosin (H&E), and observed by using light microscopy. Finally, the thrombus clearance area was calculated quantitatively by using image J. The thrombus clearance rate is calculated as follows:

$${\rm{Thrombus}}\,{\rm{clearance}}\,{\rm{rate = }}\,{\rm{\Delta }}{{\rm{W}}_{\rm{0}}}{\rm{/W \times 100\% }}$$

∆W_0_ is the area of thrombus clearance and W is the area of the vessel lumen.

#### In vivo imaging

The FeCl_3_-induced formation of rat femoral thrombosis model was established by using the same method as above. In order to screen the optimal laser irradiation time after intravenous administration in rats, we injected DiD-labeled PLT-PPy/Riv NPs in the tail vein. In vivo imaging system (IVIS) was performed at intervals of 15, 30, 45, 60, 75, and 90 min, respectively, and the fluorescence intensity was recorded, and the time of maximum fluorescence intensity was taken as the start time of laser irradiation.

Also, to investigate the photothermal efficiency of PBS, SDS-PPy/Riv NPs, PLT-PPy NPs, and PLT-PPy/Riv NPs, the rat femoral vessels were exposed to laser (808 nm, 2.22 W/cm^2^) for 10 min at 75 min after administration. The infrared thermal images and local temperature changes were recorded by infrared thermal imaging camera.

#### Tail bleeding and clotting time assessment

**Fig. 2:**
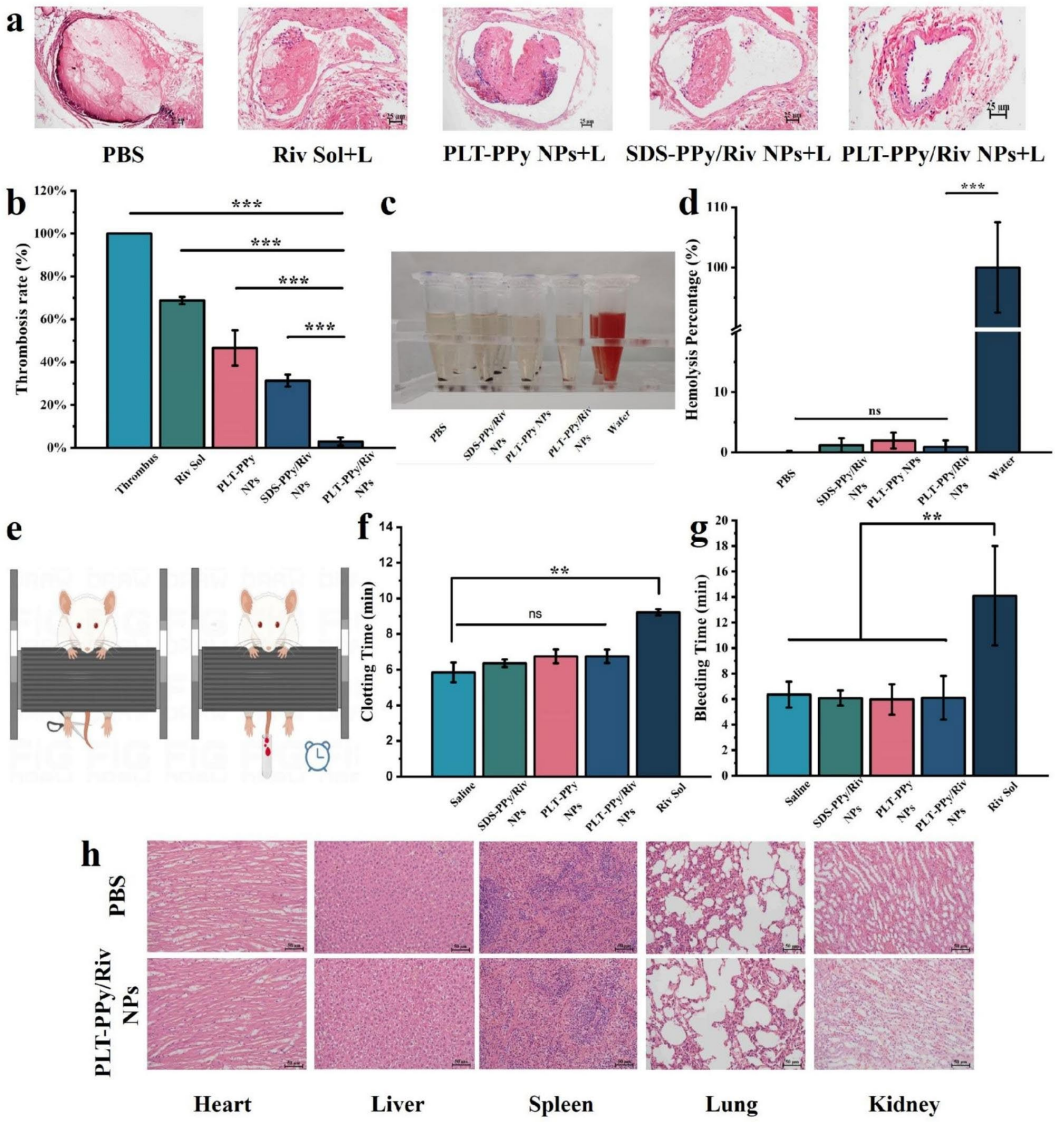
Evaluation of site-specific photothermal-amplified thrombolytic effect and safety of PLT-PPy/Riv NPs in FeCl_3_-induced rats models of LEDVT. **a** Histological microscopic analysis of rat vessels after treatment with different formulations. Scale bar = 25 μm. **b** Quantification of thrombus clearance by the ratio of thrombolytic area/total lumen area (n = 3). **c** Qualitative and quantitative **d** analysis of the hemolysis activity of SDS-PPy/Riv NPs, PLT-PPy NPs, and PLT-PPy/Riv NPs at the same Riv concentration (n = 3). **e** The mice tail-cutting model for tail bleeding test. **f** The clotting time and **g** bleeding time in the tail bleeding test (n = 3). **h** The H&E sections of heart, liver, spleen, lung and kidney of PBS and PLT-PPy/Riv NPs treatment group for evaluating the histopathological changes. Scale bar = 25 μm. Statistical analysis was via one-way ANOVA with GraphPad Prism 8.0; ns: no significance, **P < 0.01, ***P < 0.001

After anesthesia with intraperitoneal injection of chloral hydrate (7%), KM mice were injected intravenously with PBS, Riv Sol, SDS-PPy/Riv NPs, PLT-PPy NPs, and PLT-PPy/Riv NPs. After 4 h of administration, the distal end of the tail (1 cm) of mice was cut with surgical scissors (Fig. 2e). Tail bleeding time and clotting time were recorded to evaluate the anticoagulant effect. Bleeding time was defined as the time required to stop bleeding from the wound for at least 10 s [24]. The clotting time was measured through the glass slide method by taking 50 μL of the whole blood on a dry slide and starting the timing until the appearance of fibrin filaments [25].

#### Evaluation of therapeutic safety

To preliminarily assess the toxicity of the prepared preparations, the hemolysis rate of red blood cells (RBCs) in SD rats was determined. The collected blood of 1 mL of SD rats was placed in an anticoagulant tube and centrifuged at the speed of 8000 rpm for 5 min to obtain erythrocyte precipitates. 1 mL of PBS was added and the erythrocytes were washed and centrifuged several times until the supernatant was clarified. Then, the lyophilized PLT-PPy NPs, PLT-PPy/Riv NPs and SDS-PPy/Riv NPs were dissolved in PBS containing erythrocytes as the experimental group. The positive and negative control group were deionized water and PBS, respectively. After co-culture at 37 ℃ for 4 h, the mixture was centrifuged at 13,000 rpm for 10 min to remove the unbroken erythrocytes. The supernatant was taken in a 96-well plate, and the absorbance at 540 nm was measured with a microplate reader.

In addition to thrombus-containing vessels, the organs (heart, liver, spleen, lung, kidney) of different groups were took in turn, immersed in 4% paraformaldehyde, embedded, sectioned, stained with H&E and the pathological changes were observed by light microscopy.

### Statistical analysis

All data in this experiment were presented as mean ± standard deviation (SD). One-way analysis of variance (ANOVA) was performed by using GraphPad Prism to determine statistical significance. The asterisks denote the level of statistical significance (ns: no significance, *p < 0.05, **p < 0.01, ***p < 0.001 and ****p < 0.0001).

## Result and discussion

### Preparation and characterization of NPs

**Fig. 3:**
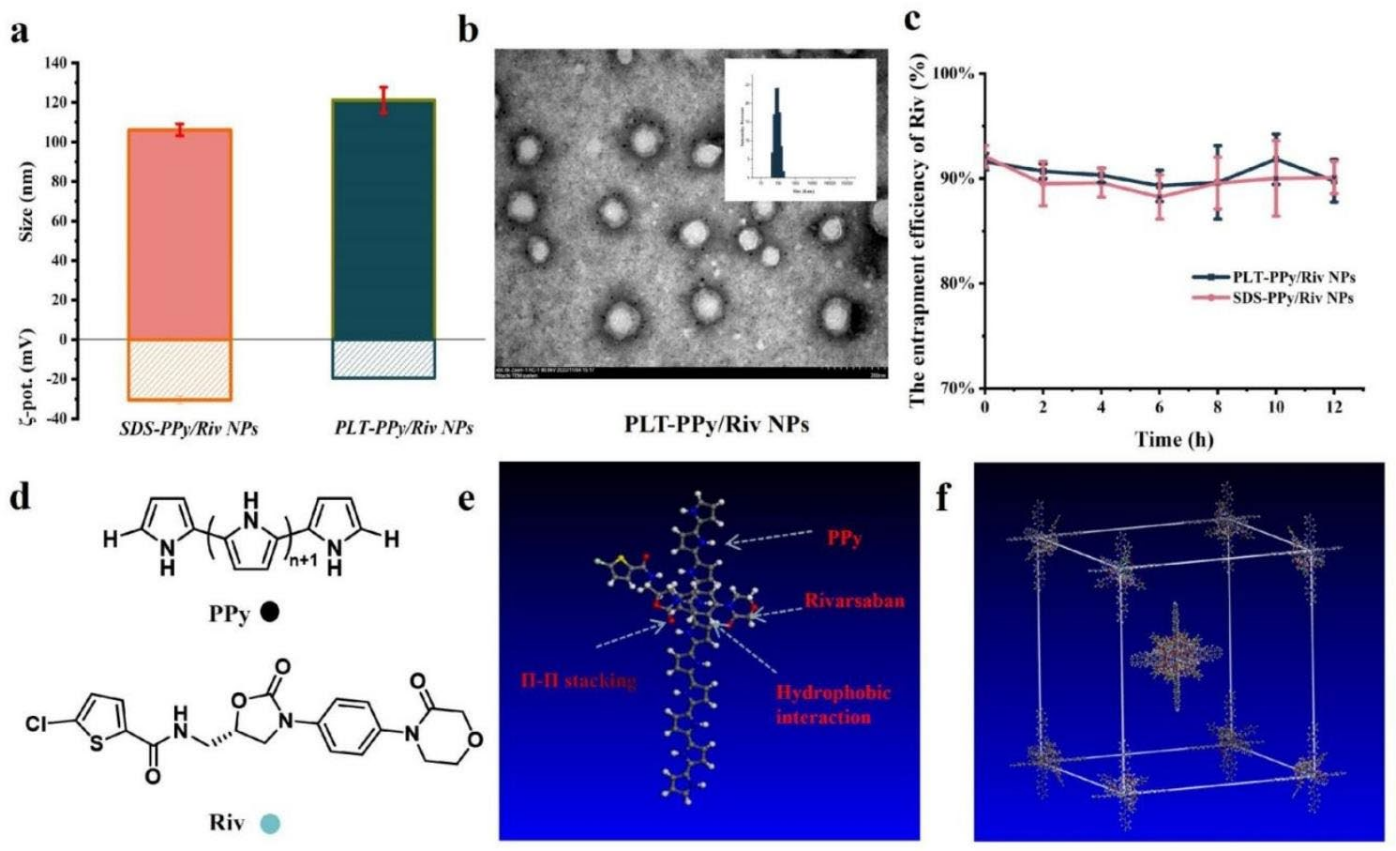
The characterization and molecular simulation of PLT-PPy/Riv NPs. **a** Physicochemical properties of SDS-PPy/Riv NPs and PLT-PPy/Riv NPs (n = 3). ζ- pot: Surface charge. **b** Particle-size distribution profile and TEM image of PLT-PPy/Riv NPs. **c** Riv leakage of SDS-PPy/Riv NPs and PLT-PPy/Riv NPs in PBS (pH 7.4) containing 10% FBS incubated at 37 °C for 12 h. **d** The chemical structures of PPy and Riv. **e** Schematic diagram of molecular simulation of PPy and Riv. **f** The three-dimensional crystal cell structure of PLT-PPy/Riv NPs

SDS-PPy/Riv NPs were prepared by chemical oxidation polymerization, and the platelet membrane was successfully coated on the surface of SDS-PPy/Riv NPs by ultrasound and extrusion. The particle size of the prepared SDS-PPy/Riv NPs and PLT-PPy/Riv NPs were 106.1 and 121.1 nm, respectively, and the zeta potentials were − 30 mV and − 19 mV, respectively, when characterized by Zetasizer (Fig. 3a). The PLT-PPy/Riv NPs had an increase in particle size of about 15 nm and had the same surface charge as that of platelet membranes, providing preliminarily evidence of successful coating of the platelet membranes (Table 1). Observed by TEM with a voltage of 80 kV, the PLT-PPy/Riv NPs were spherical with good dispersion and obvious core-shell structure (Fig. 3b). When optimizing the entrapment efficiency (EE) of Riv, the mass ratio of Py and Riv had a significant impact on the EE of Riv. By ultrafiltration method, the EE of Riv in PLT-PPy/Riv NPs was calculated by HPLC as high as 91.1%. Meanwhile, as shown in Fig. 3c, more than 90% of Riv could be stably maintained in PBS containing 10% FBS for 12 h at 37 °C. These results indicated that SDS-PPy/Riv NPs and PLT-PPy/Riv NPs had good co-loading stability in the presence of salt and protein.

**Table 1:** Physiochemical characteristics of the prepared nano-formulations

Formulations	Size^a^ (nm)	PDI^b^	Zeta potential^a^ (mV)	EE^c^ (Riv)
SDS-PPy/Riv NPs	106.1 ± 2.1	0.186	-30 ± 0.9	93.6%
PLT-PPy/Riv NPs	121.1 ± 0.9	0.172	-19 ± 1.7	91.1%

^a)^ Mean diameters and Zeta potential of nanoparticles were determined by Zetasizer.^b)^ Polydispersity index of nanoparticles size.^c)^ EE was calculated by the following equation:

$$EE\left(\%\right)=\frac{{W}_{2}-{W}_{1}}{{W}_{2}}\times 100\%$$

Where W_1_ stands for the amount of Riv in the filtrate and W_2_ is the total amount.

It was hypothesized that the successful loading of Riv was due to the Π-Π stacking interactions between PPy and Riv molecules, which also promoted the formation of SDS-PPy/Riv NPs under the action of amphiphilic SDS. In order to explore their mode of action, we performed molecular dynamics to calculate the binding energy of PPy and Riv, and screen the degree of pyrrole polymerization when PPy and Riv reached the optimal binding energy. The result of calculation was shown in Table 2: the binding energy of PPy and Riv was gradually increasing when the pyrrole monomer was polymerized from 1 to 10, and the binding energy of PPy and Riv was gradually weakening when n = 10 to 20. When n = 10, the binding effect of PPy and Riv was the strongest. The results of molecular simulation were showed in Fig. 3d and e, and the main forces of PPy and Riv were stacking and intermolecular hydrophobic interaction force. Finally, we preliminary constructed and optimized the three-dimensional crystal cell of PPy bound to Riv as in Fig. 3f.

**Table 2:** Calculation results of the binding energies of PPy with different polymerization degrees and Riv (kcal/mol)

Pairs	n = 2	n = 4	n = 6	n = 8	n = 10	n = 12	n = 14	n = 16	n = 18	n = 20
PPy-PPy	-2.26	-8.46	-15.46	-20.43	-24.14	-28.58	-37.60	-38.59	-45.52	-47.40
Riv-Riv	-12.42	-12.83	-12.64	-12.85	-12.72	-15.47	-12.52	-12.03	-12.52	-11.20
PPy-Riv	-5.23	-6.92	-8.52	-9.089	-11.39	-9.01	-9.81	-9.19	-9.43	-9.86

### In vitro photothermal efficiency and drug release of PLT-PPy/Riv NPs

**Fig. 4:**
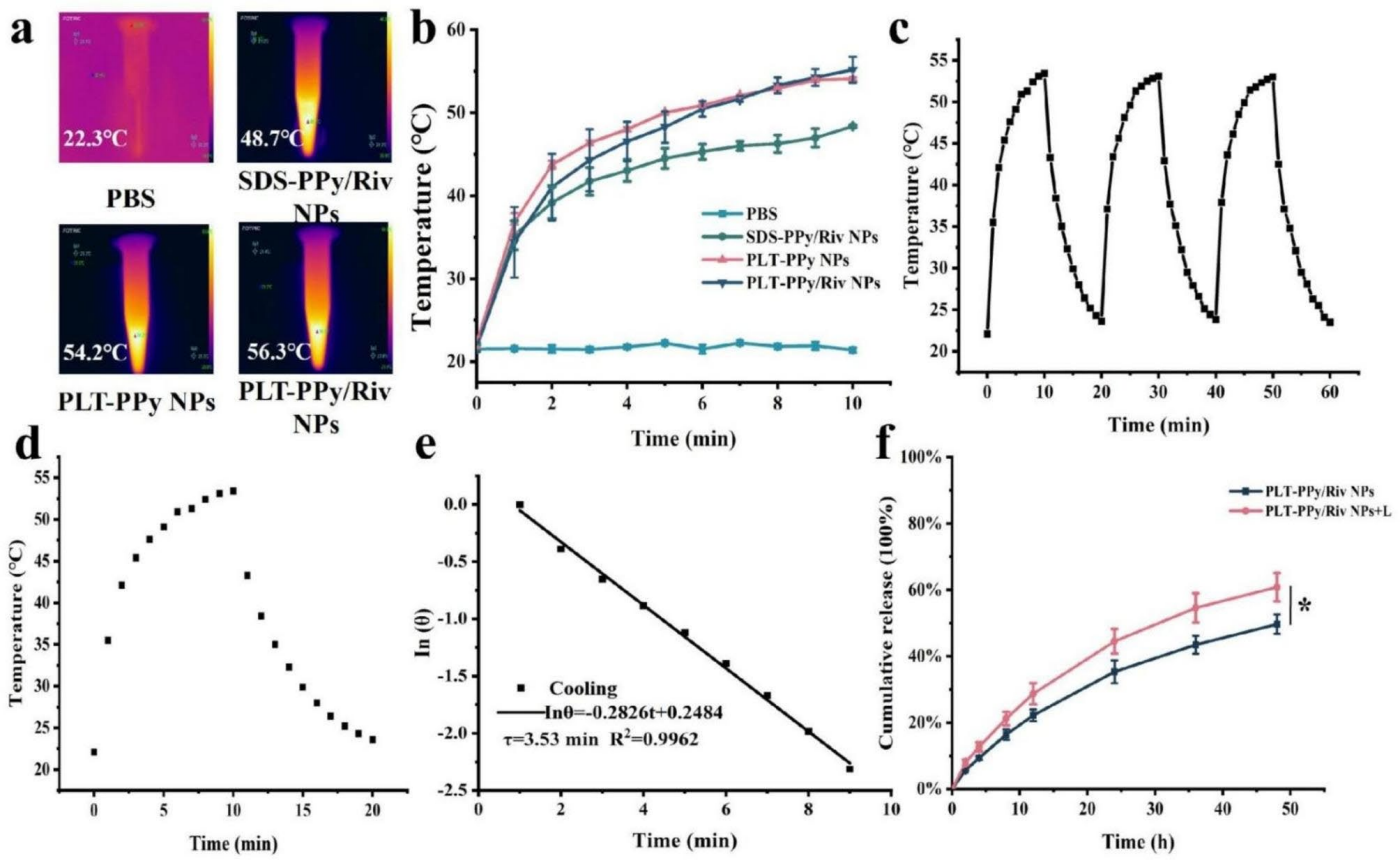
In vitro photothermal conversion efficiency of PLT-PPy/Riv NPs. **a** In vitro thermal imaging and (**b**) temperature change of PBS, SDS-PPy/Riv NPs, PLT-PPy NPs and PLT-PPy/Riv NPs under 808 nm laser irradiation within 10 min (n = 3). **c** The photothermal stability of PLT-PPy/Riv NPs under NIR irradiation using the 808 nm laser (2.22 W/cm^2^, 1200 s for each cycle) for three cycles. **d** Natural cooling curve of PLT-PPy/Riv NPs under laser irradiation. **e** Linear fit of time-Inθ from the cooling period of PLT-PPy/Riv NPs. **f** In vitro release of Riv from PLT-PPy/Riv NPs under laser or non-laser irradiation. Statistical analysis was via one-way ANOVA with GraphPad Prism 8.0; *P < 0.05

To investigate the effect of platelet membrane encapsulation process on the photothermal performance of the inner core NPs SDS-PPy/Riv NPs, we used 808 nm laser to irradiate PBS, SDS-PPy/Riv NPs, PLT-PPy NPs, PLT-PPy/Riv NPs for 10 min and then plotted the temperature increase curves. The results were shown in Fig. 4a, b. It was found that SDS-PPy/Riv NPs could reach a maximum of 48.4 ℃ within 10 min, while the maximum temperatures of PLT-PPy NPs and PLT-PPy/Riv NPs were 54.2 ℃ and 55.1 ℃, respectively, which could successfully cause the thermal disintegration of blood clots. The membrane-encapsulated NPs did not interfere with the photothermal effect of the inner NPs. Instead, the highest temperature of the membrane-coated NPs was higher than that of the inner NPs. To calculate the PCE of PLT-PPy/Riv NPs, we recorded the natural cooling variation of PLT-PPy/Riv NPs and plotted the cooling curve in Fig. 4d. The results of time-Inθ linear fitting were showed in Fig. 4e and the time constant τ is 211.8 s. After calculation, hs was 19.83 mW/℃, the maximum stable temperature Tmax was 56.6 ℃, and the ambient temperature Tsur was 22 ℃. And the ultraviolet absorbance A of PLT-PPy/Riv NPs at 808 nm was 3.91, I was the laser power 2.22 W/cm^2^, and Qs was the heat loss of water 0.3966 mW. Therefore, the PCE of PLT-PPy/Riv NPs was 30.86%. The results showed that PLT-PPy/Riv NPs had sufficiently high PCE and photothermal stability (Fig. 4c). The increase of temperature was helpful to the ablation of thrombus to achieved good therapeutic effect.

Furthermore, Fig. 4f showed that the cumulative release of Riv in PLT-PPy/Riv NPs without laser irradiation was 49.66% at 48 h, whereas the laser-irradiated Riv was able to release about 60%, and there was certain significant difference between them. It was speculated that the laser-mediated high temperature-induced weakened of the intermolecular forces of Riv in PLT-PPy/Riv NPs, and thus PLT-PPy/Riv NPs had some photo-thermal responsive drug release performance.

### In vitro evaluation of thrombosis targeting ability

**Fig. 5:**
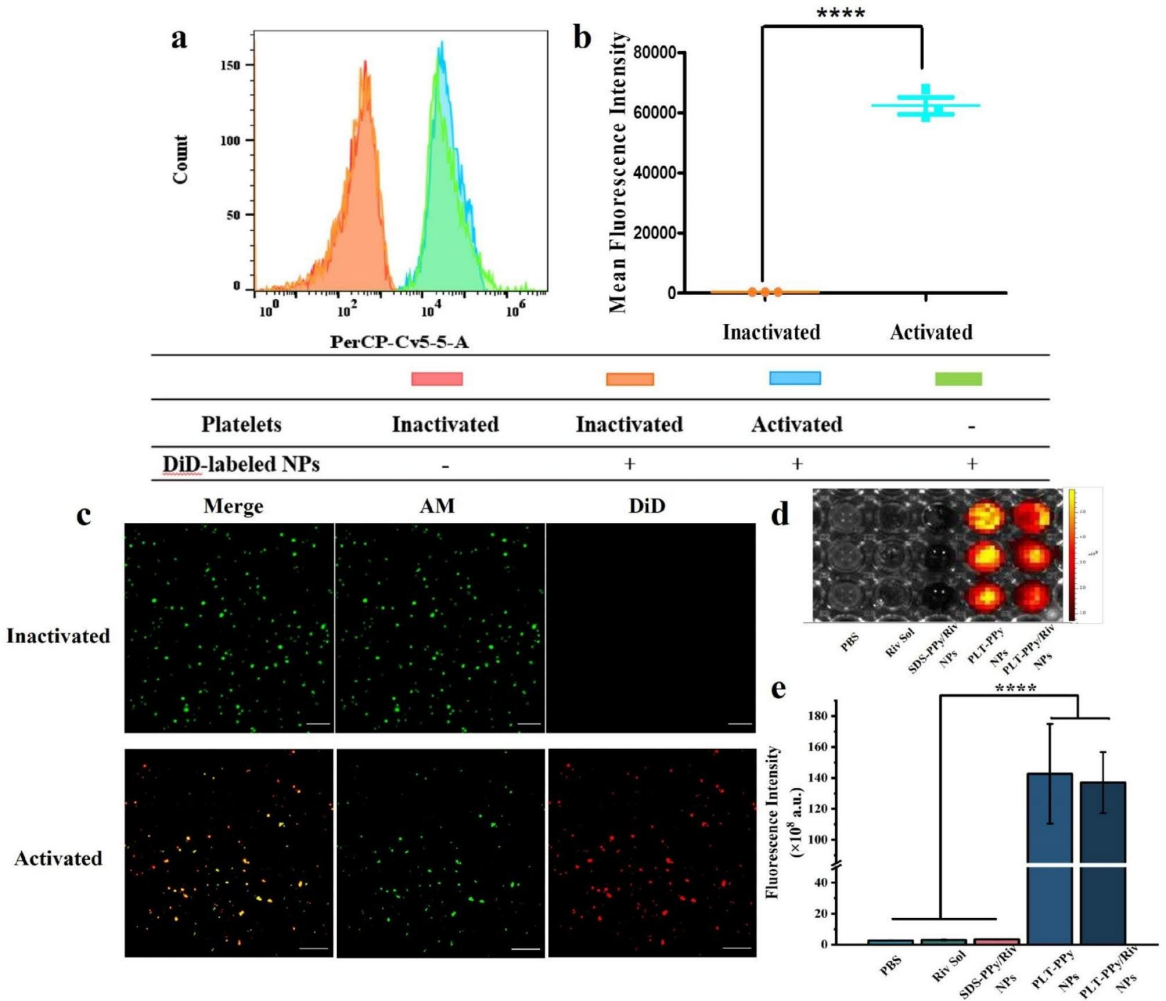
In vitro assessment of thrombus-targeting capabilities of PLT-PPy/Riv NPs. **a** Flow cytometry analysis of the adhesion of PLT-PPy/Riv NPs to activated and inactivated platelets and **b** quantitative analysis of fluorescence intensity (n = 3). **c** The adhesion of PLT-PPy/Riv NPs to activated and inactivated platelets observed by laser confocal microscopy. Platelets were stained with calcein-AM (green) and PLT-PPy/Riv NPs were labeled with DiD (red). Scale bar = 10 μm. **d** In vitro fluorescence signal in artificial blood clots (n = 3). **e** Quantitative analysis of fluorescence intensity in artificial blood clots (n = 3). Statistical analysis was via one-way ANOVA with GraphPad Prism 8.0; ****P < 0.0001

After determining that the membranes were successfully coated on the polymer surface, we performed an in vitro targeting evaluation to explore the potential of biomimetic NPs for thrombotic adhesion. Activated platelets are one of the major components of thrombus. Here, we assessed the binding ability of PLT-PPy/Riv NPs to activated platelets by flow cytometry analysis. In the flow cytometry assay (Fig. 5a, b), a right shift of the fluorescence peak and a significant enhancement of the fluorescence intensity were observed in both the activated and positive control groups compared with the inactivated group. These indicated that DiD-labeled PLT-PPy/Riv NPs preferentially bounded only to activated platelets, while no false positives were observed. Meanwhile, the fluorescence intensity in the inactivated group was similar to that in the negative control group, indicating that DiD-labeled PLT-PPy/Riv NPs in the inactivated group had not yet bounded to inactivated platelets. The adhesion of PLT-PPy/Riv NPs to activated platelets was further verified and visualized using confocal microscopy (Fig. 5c). The overlap of red fluorescence of PLT-PPy/Riv NPs and green fluorescence of platelets in the activated group confirmed the successful adhesion of DiD-labeled PLT-PPy/Riv NPs to the surface of activated platelets, and further demonstrated the successful coating of platelet membranes in the bionanodelivery system.

In addition, we also prepared artificial blood clots to investigate the thrombus targeting ability of PLT-PPy/Riv NPs in vitro. Platelet membrane-coated PLT-PPy NPs and PLT-PPy/Riv NPs showed significantly stronger fluorescence intensity than PBS, Riv Sol and SDS-PPy/Riv NPs in blood clots, indicating that platelet membranes played an important role in thrombus targeting (Fig. 5d, e).

### In vitro clot lysis test

**Fig. 6:**
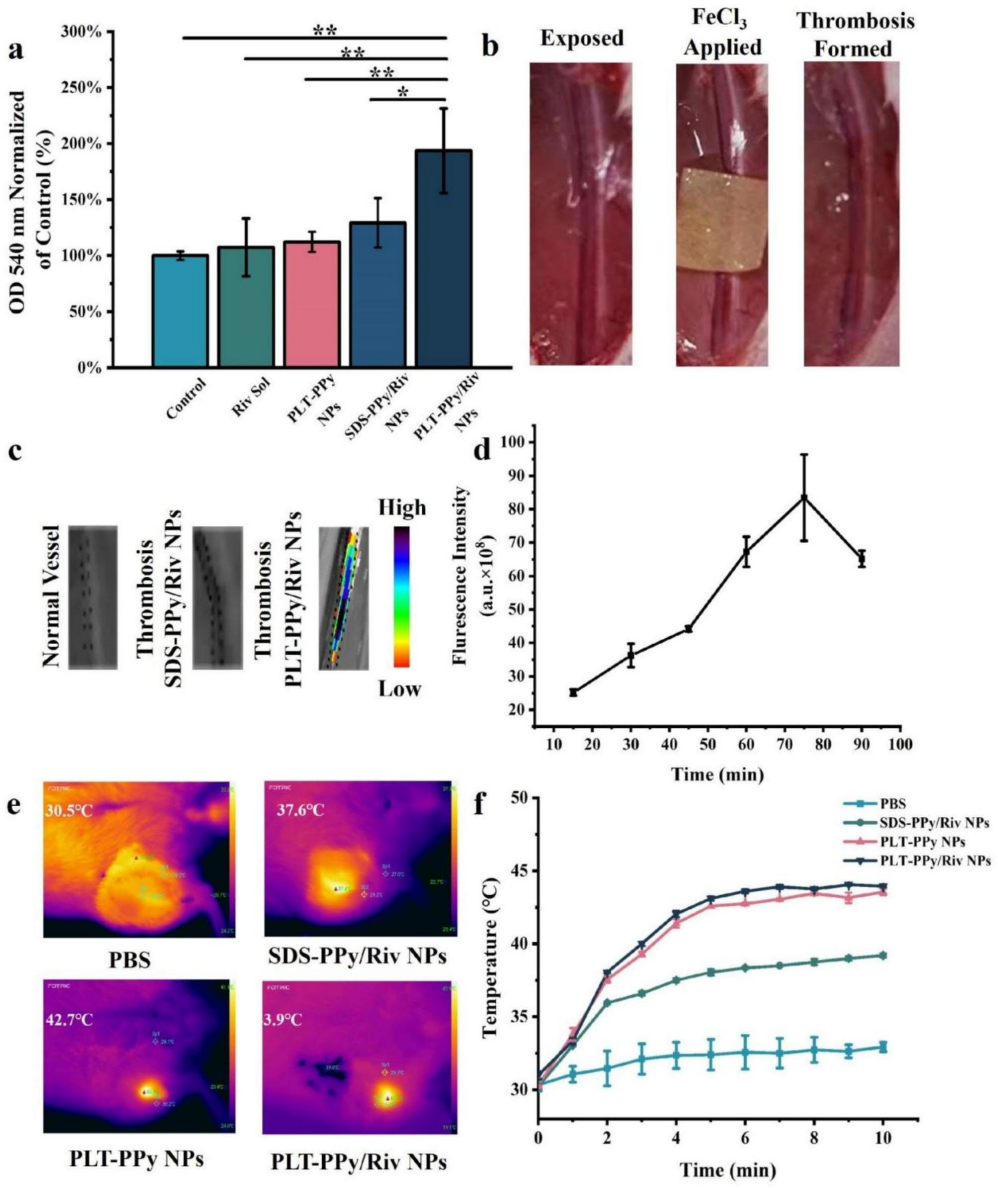
In vitro clot lysis and in vivo thrombus targeting assessment of PLT-PPy/Riv NPs. **a** Quantitative analysis of in vitro thrombolysis of PBS, Riv Sol, SDS-PPy/Riv NPs, PLT-PPy NPs and PLT-PPy/Riv NPs after laser irradiation for 10 min. **b** Thrombosis model in animals induced by ferric chloride method. **c** In vivo fluorescence images of femoral vein vessels at 75 min post-injection of PLT-PPy/Riv NPs collected by using IVIS. **d** Quantitative analysis of the fluorescence intensity changes of DiD-labeled-PLT-PPy/Riv NPs at different time points after intravenous injection (n = 3). **e** In vivo photothermal imaging and **f** in vivo temperature change curves under 808 nm laser irradiation after administration of different formulations. Statistical analysis was via one-way ANOVA with GraphPad Prism 8.0; *P < 0.05, **P < 0.01

Here, the Drabkin method was used to assess the in vitro clot lysis effect of PLT-PPy/Riv NPs. The principle of the Drabkin test method refered to the interaction of the Drabkin’s reagent with hemoglobin or erythrocytes released from lysed clots, which was converted into cyanogenic hemoglobin, and could be quantified by determining the absorbance value of the hemoglobin at 540 nm [26]. In general, with the increase of antithrombotic degree, the clots would release more red blood cells, and the absorbance of the supernatant measured at 540 nm would increase. As shown in Fig. 6a, the in vitro clot lysis effects after treatment were, in descending order, PLT-PPy/Riv NPs > SDS-PPy/Riv NPs > PLT-PPy NPs > Riv Sol. The therapeutic effect of PLT-PPy/Riv NPs was 1.8-fold that of single-drug therapy, 1.72-fold that of single-photothermal therapy, and 1.5-fold that of untargeted therapy. These results indicated that local hyperthermia could effectively enhance the antithrombotic effect of Riv, which implied that the prepared PLT-PPy/Riv NPs could perform light-driven hyperthermia with Riv activity, and ultimately implemented effective antithrombotic therapy.

### In vivo imaging

Based on the principle of redox-induced endothelial cell damage, ferric chloride is widely used in the modeling of animal thrombosis models. In order to study the treatment of thrombosis in vivo, we used ferric chloride to cover the femoral vein to induce thrombus formation and the results were showed in Fig. 6b. The blood vessels changed from bright red to dark red accompanied by vasoconstriction, representing successful thrombosis [27, 28].

To screen the optimal laser-irradiation time after tail vein administration, the time of the strongest fluorescence intensity at the thrombus site after administration was taken as the time of laser irradiation. As shown in Fig. 6c, d, it showed significant fluorescence intensity at the vascular embolization site at 75 min, implying that PLT-PPy/Riv NPs were most enriched in thrombus at 75 min. Therefore, 75 min was regarded as the optimal time for 808 nm laser irradiation after nano-administration of PLT-PPy/Riv NPs.

Next, we evaluated the in vivo photothermal properties of PLT-PPy/Riv NPs in FeCl_3_-induced femoral vein thrombosis models. The 808 nm laser was used to specifically irradiate the thrombosis site at 75 min post-administration. As shown in Fig. 6e, f, after 10 min of continuous irradiation, the temperature of the thrombus site in the PLT-PPy/Riv NPs group reached 44℃, the in vivo PCE of which was similar to that of PLT-PPy NPs (about 43℃). However, SDS-PPy/Riv NPs group (about 37℃) exerted poor thrombus targeting and low enrichment in the thrombus, resulting in a slightly lower in vivo PCE than that of PLT-PPy/Riv NPs and PLT-PPy NPs group. Fibrin clots were found to be heat-sensitive, and the clots disintegrated when the temperature was increased to 42℃~50℃ [8]. Therefore, compared with PLT-PPy NPs and SDS-PPy/Riv NPs, PLT-PPy/Riv NPs could be used as an excellent antithrombotic agent and showed optimal thrombolytic effects due to their own thrombus-targeting and drug loading characteristic.

### In vivo thrombosis therapeutic efficacy and safety studies

Different formulations were injected into SD rats of femoral vein thrombosis model to assess the in vivo targeted antithrombotic effect. Figure 2a showed the H&E section images of the femoral vein containing thrombus after different treatments, and Fig. 2b showed the thrombus clearance rate calculated based on the above H&E images. From these results, it was obvious that the in vivo therapeutic effects, in descending order, were as follows: PLT-PPy/Riv NPs > SDS-PPy/Riv NPs > PLT-PPy NPs > Riv Sol. The Riv Sol group had a slight antithrombotic effect due to the rapid clearance and off-target effect of free Riv in vivo. The PLT-PPy NPs group with laser irradiation only had a more favorable effect than the free Riv group. And the Riv Sol group with laser irradiation had a better antithrombotic effect than that without laser irradiation, suggesting that localized photothermal therapy at the thrombus site could enhance the antithrombotic effect to some extent. Meanwhile, laser irradiation to the SDS-PPy/Riv NPs group with no off-target effect enhanced the thrombolytic effect, implying a synergistic effect of photothermal therapy and drug therapy. Compared with the SDS-PPy/Riv NPs group and the PBS group, the PLT-PPy/Riv NPs group showed a significant antithrombotic effect with a thrombolytic rate of up to 90%, which was attributed to the selective aggregation of Riv at the thrombus site and the combined effect of localized photothermia.

We further evaluated the in vivo anticoagulant effect of PLT-PPy/Riv NPs by tail bleeding assay in KM mice. PBS, Riv Sol, SDS-PPy/Riv NPs, PLT-PPy NPs and PLT-PPy/Riv NPs were injected intravenously, respectively. After 4 h, the tail of mice was removed and then the timing started after bleeding, and the bleeding time and clotting time were used as the evaluation index of anticoagulant ability. Mice have inherent hemostasis, as shown in Fig. 2f, g, rapid hemostasis and coagulation occurred in PBS group within 6 min. SDS-PPy/Riv NPs, PLT-PPy NPs, PLT-PPy/Riv NPs showed no significant difference in their bleeding time and clotting time compared with the PBS group due to good thrombus targeting ability and slow release characteristics of Riv. However, free Riv significantly prolonged the bleeding time and clotting time due to systemic off-targeting. In addition, the bleeding time of PLT-PPy/Riv NPs and PLT-PPy NPs was not significantly different from that of the control group, and no significant bleeding risk was detected, suggesting that the body’s return to normal coagulation was not affected by NPs.

The hemolysis and pathological sections of major organs were performed to preliminary evaluated the in vivo toxicity of PLT-PPy/Riv NPs. As shown in Fig. 2c, d, the hemolysis rate of PLT-PPy/Riv NPs was less than 5%, indicating that there was no potential hemolytic risk in the treated rats. As shown in Fig. 2h, compared with the control group, no obvious histopathological changes, including necrosis, fibrosis and hydropathy, were observed in the pathological sections of heart, liver, spleen, lung and kidney in the PLT-PPy/Riv NPs treatment group. All these experimental results proved that PLT-PPy/Riv NPs had good in vivo compatibility, indicating that PLT-PPy/Riv NPs were safe for use in vivo and in the treatment of thrombotic diseases.

## Conclusions

In this study, Riv-loaded PPy NPs were prepared by chemical oxidative polymerization method. The negatively charged drug-loaded PPy NPs were prepared by using the “bottom-up” surface nanoengineering technique. And platelet membranes were “right-side-out” structure were obtained by electrostatic repulasion principle, so that the platelet membrane proteins were retained and oriented outward-side. Finally, The PLT-PPy/Riv NPs with platelet-like biological functions were successfully prepared. The binding of Riv to PPy was based on Π-Π stacking and intermolecular hydrophobic interactions, which successfully realized the drug loading of PPy NPs while retaining the original photothermal properties of PPy NPs. In addition, the spectificlly targeting principle of platelet membranes to the thrombus site was utilized to promote precise enrichment of PLT-PPy/Riv NPs at thrombus site to achieve high thrombus clearance rate. As thrombus-targeted thrombolytic nanomedicine, PLT-PPy/Riv NPs had a high safety. Since the internal protein of the extracted platelets had been largely cleared, they couldn’t activate the downstream pathway of native platelets, induce platelet activation and aggregation, or exhibit thrombogenic activity. Moreover, we used hypothermia as an adjuvant treatment to avoid local high temperature damage to normal tissues, which had great application potential and research value in the direction of photothermal targeted thrombolysis.

Several major challenges faced in thrombosis therapy were addressed: increasing drug enrichment at the thrombus site, reducing the bleeding risk of anticoagulants, and reducing drug clearance in the blood. However, PLT-PPy/Riv NPs still have some technical difficulties to be broken through in their application to the clinic: (1) Safety: Although PLT-PPy/Riv NPs have shown good safety in the laboratory research stage, they face the same clinical translational issues as traditional nano-targeted drug delivery systems, such as the degradation safety of the polymer materials, which is still the difficulty in the development of an efficient and safe nano-thrombotic drug delivery systems. (2) Immunogenicity: PLT-PPy/Riv NPs administration is similar to platelet transfusion, and there are potential immunogenicity problems with platelets from different sources. Using patient-derived, matched platelets and high-purity platelets or undergo sensitization testing will provied reference for clinical administration of PLT-PPy/Riv NPs. (3) Laser penetration depth: Compared with traditional therapies, the localized photothermolysis by 808 nm laser has high selectivity, short treatment time and obvious efficacy. Within the range of the human-body optical window, 808 cm can non-invasively penetrate the human-body subcutaneous tissues to a depth of 5–7 cm, while cannot penetrate into the deep tissues of the human body. Therefore, it is required exposed bloods vessels for thermotherapy in LEDVT. There are some limitations to the treatment of diseases inside the body. Combined with these considerations, antithrombotic bionic drug delivery systems such as PLT-PPy/Riv NPs will be closer to clinical practice in the foreseeable future.

## Data Availability

The date supporting this study’s fingings are available within the paper.

## Abbreviations

ACD: Acid-citrate-dextrose
ANOVA: One-way analysis of variance (ANOVA)
DiD: 1,1′-dioctadecyl-3,3,3′,3′-tetramethylindodicarbocyanine, 4-chlorobenzenesulfonate salt dye
FBS: Fetal bovine serum
H&E: Hematoxylin and eosin
HPLC: High performance liquid chromatography
IVIS: In vivo imaging system
KM mice: Kunming mice
LEDVT: Lower extremity deep venous thrombosis
NPs: Nanoparticles
PBS: Phosphate buffered saline
PCE: Photothermal conversion efficiency
PGE1: Prostaglandin 1
PLT: Platelet
PMSF: Phenylmethanesulfonyl fluoride
PPy: Polypyrrole
PRP: Platelet-rich plasma
PTT: Photothermal therapy
RBCs: Red blood cells
Riv: Rivaroxban
SD rats: Sprague-Dawley rats
SDS: Sodium dodecyl sulfate
TEM: Transmission electron microscopy
